# Spatiotemporal changes in the distribution of LHFPL5 in mice cochlear hair bundles during development and in the absence of PCDH15

**DOI:** 10.1371/journal.pone.0185285

**Published:** 2017-10-25

**Authors:** Shanthini Mahendrasingam, Robert Fettiplace, Kumar N. Alagramam, Ellen Cross, David N. Furness

**Affiliations:** 1 School of Life Sciences, Keele University, Keele, Staffordshire, United Kingdom; 2 Department of Neuroscience, University of Wisconsin School of Medicine and Public Health, Madison, Wisconsin, WI, United States of America; 3 Otolaryngology Head and Neck Surgery, University Hospital Cleveland Medical Center, Case Western Reserve University, Cleveland, Ohio, OH, United States of America; Universidad de Salamanca, SPAIN

## Abstract

Mechanosensory transduction by vertebrate hair cells depends on a protein complex at the tips of shorter stereocilia associated with mechanoelectrical transduction channels activated by tip links in the hair bundle. In mammalian hair cells, this complex includes transmembrane channel-like protein subunit 1 (TMC1), lipoma HMGIC fusion partner-like 5 protein (LHFPL5) and protocadherin 15 (PCDH15), a lower-end component of the tip link. TMC1 interacts with LHFPL5 and PCDH15 but how the complex develops to maturity, and the relationships between these proteins, remains uncertain. Here we evaluate the spatiotemporal development of LHFPL5 distributions in mouse cochlear hair bundles by immunofluorescence and immunogold transmission electron microscopy, from postnatal day 0 (P0) through P21 in wild type and PCDH15-deficient mice. At P0, hair bundles contain many short microvilli-like processes which we term unranked stereocilia, and a subset of lengthening rows, adjacent to a kinocilium. LHFPL5 is distributed throughout the bundle, including on stereocilia tips and the kinocilium. At P3, 4-to-6 rows of ranked stereocilia are evident, total LHFPL5 expression peaks, and LHFPL5 is localised to ranked stereocilia tips of all rows and to lower shaft/ankle links. By P12, the bundle has a mature pattern with 3 ranked rows but virtually no unranked stereocilia or kinocilium; LHFPL5 expression has declined and become restricted to the tips of shorter stereocilia. Throughout development from P0, expression of LHFPL5 is greater overall on apical than basal bundles, but there is, on average, an equal amount of labelling per labelled tip. In P3 mice lacking PCDH15, LHFPL5 labelling is not at the tips but is primarily on unranked stereocilia and lower lateral links. These data show that LHFPL5 is already present in the MET apparatus at P0 but requires PCDH15 at P3 to remain there. Shaft/ankle link localisation suggests it interacts with link proteins other than PCDH15.

## Introduction

Mechanoelectrical transduction (MET) channels in vertebrate hair cells are gated by a fine extracellular filament, the tip link, that is located in the sensory stereociliary bundle (see review [1]). The stereocilia are arranged in rows of increasing height and each individual stereocilium in a shorter row is connected by a single tip link to the side of the adjacent taller stereocilium in the row behind [2,3]. In normal function, the MET channel is localised to the short stereocilia tips [4] and, increasing tension on the tip link in response to deflections of the hair bundle towards the tallest row of stereocilia gates the MET channel by means of a molecular complex. Opposing deflections relax the link and the MET channel closes; the hair cell thus shows polarity in response to bundle deflections.

The tip link is composed of dimers of cadherin 23 (CDH23) forming its upper portion [5,6] and dimers of protocadherin 15 (PCDH15) forming its lower portion [6,7]. In PCDH15-deficient *Ames waltzer av3J/av3J* (*Pcdh15*^*av3J/av3J*^) or CDH23-deficient *waltzer* (*Cdh23*^*v2J/v2J*^) mice, there is a reduced number of tip links, the hair bundle is dysmorphic and hair cells show anomalous, reversed polarity transducer currents [8]. Anomalous currents also occur following BAPTA treatment in wild-type mice, which is known to destroy the tip links [9] and can be evoked by stimulating the hair cell apex during development up to postnatal day (P) 2–3 in apical inner or outer hair cells [10]. This current diminishes in immature hair cells as the normal MET current develops and is independent of the putative subunit proteins of the MET, trans-membrane-like channel protein isoforms 1 (TMC1) or 2 (TMC2), as it occurs in *Tmc1/Tmc2* knockouts [10,11]. Recent evidence suggests that the anomalous reverse-polarity currents flow through PIEZO2, mechanically sensitive channels on the hair cell apical surface [12].

Lipoma HMGIC fusion partner-like 5 (LHFPL5), also known as tetraspan membrane protein of hair cell stereocilia (TMHS) encoded by the DFNB67 gene, is reported to be interact with both PCDH15 and TMC1. LHFPL5 is required for proper targeting of both to the stereocilia tips [13,14] and has been reported to be absent from *Pcdh15*-deficient mice. It may act as the link between the PCDH15 and the MET channel [13]. Another protein of currently unknown function that is associated with both PCDH15 and LHFPL5, is TMIE (transmembrane inner ear protein) [15].

To test the hypothesis that LHFPL5 becomes associated concurrently with developmental acquisition of normal polarity transduction and the MET apparatus we evaluated the spatiotemporal changes in distribution of LHFPL5 using immunofluorescence and immunogold techniques to provide a high resolution, semi-quantitative analysis. In addition, preliminary immunofluorescence data suggested that *Pcdh15*-deficient mouse hair bundles are not completely devoid of LHFPL5, unlike the previous reports [13]. We therefore wanted to assess, with high resolution immunogold for transmission electron microscopy (TEM), whether LHFPL5 was eliminated from the tip region in the absence of the tip link in PCDH15-deficient *Pcdh15*^*av3J/av3J*^ mice. Scanning electron microscopy (SEM) was also used to provide a structural context for the ultrastructural localisation of LHFPL5 at specific locations through the developmental process. This was necessary because although a number of studies have reported development of hair bundles in a variety of species and locations (see for example [16,17]), no definitive data have been collected at high resolution at different ages from mouse at the specific location where we have performed our analyses.

## Materials and methods

### Animals

CD/1 and C3HeB/FeJ mice were used to evaluate normal distributions of LHFPL5 in the hair bundles from two different strains of mice. CD/1 mice were used to study developmental changes from P0 –P21, spanning the postnatal development period to weaning. Wild type CD/1 mice were bred and maintained in Keele University’s Central Animal facility and C3HeB/FeJ (fixed samples kindly provided by Prof Walter Marcotti) in Sheffield University animal unit. All animal maintenance and treatment of these mice during preparation was under the UK Animals (Scientific Procedures) Act of 1986. *Lhfpl5* heterozygous (*Lhfpl*5^+/-^) and homozygous (*Lhfpl*5^-/-^) mutant mice were used at P5 and P12 to check for labelling specificity (B6.129-Lhfpl5tm1Kjn/Kjn; The Jackson Laboratory). *Lhfpl*5^+/-^and *Lhfpl*5^-/-^ mice were maintained at the University of Wisconsin-Madison. Their maintenance and sample preparation were performed using methods approved by the Institutional Animal Care and Use Committee of the University of Wisconsin-Madison. *Pcdh15* mutants heterozygous (*Pcdh15*^+*/av3J*^) and homozygous (*Pcdh15*^*av3J/av3J*^) mice were bred at Case Western Reserve University (CWRU) and their use was approved under protocol number 2010–0074 (entitled ‘Characterization of mouse models of deafness’) by the Institutional Animal Care and Use Committee (IACUC). For all the genotypes and genetic background indicated here, inner ear tissue was obtained from both male and female mice.

### Scanning electron microscopy

To evaluate the 3D bundle morphology during development, we examined mouse cochlear hair cells at different developmental stages. Cochleae of CD/1 mice aged P0 to P10 were anaesthetised with an overdose of sodium pentobarbitone (IP; Pentoject, Animalcare Ltd, York), decapitated, the cochleae removed and fixed through holes in the round window and the apex, with 2.5% glutaraldehyde (GTA) in 0.1 sodium cacodylate buffer (NaCac) for 2 h. They were dissected into spirals, post-fixed in 1% OsO_4_/NaCac and then osmium-thiocarbohydrazide impregnated using the OTOTO technique [3,18]. They were subsequently dehydrated through an ethanol series and critical point dried from liquid CO_2_, before being mounted on sticky carbon pads onto SEM stubs. Where necessary, to reduce charging, the specimens were further grounded using silver colloid paint (Agar Scientific) and examined in a Hitachi S4500 field emission SEM operated at 5kV. We did not use gold-labelling with backscattered electron detection for SEM because preliminary results showed that much of the labelling was located developmentally in the lower part of the bundle, between stereocilia, and therefore not accessible to observation in an SEM.

### Immunolabelling

#### Antibodies

The primary antibody was an affinity-purified rabbit polyclonal antibody to the C-terminal region of human LHFPL5 predicted to recognise both mouse and rat (Cat# OAAB00424, Aviva Systems Biology, San Diego, USA). The secondary antibodies were Alexa Fluor 568 goat anti-rabbit IgG (H+L) (Invitrogen Molecular Probes, Oregon, USA), and goat anti-rabbit IgG conjugated to 10 nm gold particles (British BioCell, Cardiff, UK). Phalloidin-fluorescein isothiocyanate (phalloidin-FITC; P5282; Sigma-Aldrich) was used to detect actin and visualise the stereocilia.

#### Fixation

CD/1 and C3HeB/FeJ mice were anaesthetised with an overdose of sodium pentobarbitone (IP; Pentoject, Animalcare Ltd, York), decapitated, the cochleae removed and fixed through holes in the round window and the apex, with 4% paraformaldehyde (PFA) in 0.1M sodium phosphate buffer (PB) or 4% PFA + 0.1% glutaraldehyde (GTA) in PB for 1–2 h at room temperature. The samples were stored in 1/10th concentration of the fixative used for fixation and kept at 4°C until further processing and immunolabelling. *Lhfpl*5^+/-^and *Lhfpl*5^-/-^mice were killed by decapitation, fixed by perfusion with 4% PFA in PB, pH 7.4, through the round window and a small hole made in the apex and immersed in the same fixative for 1h. The *Pcdh15*^+*/av3J*^ and *Pcdh15*^*av3J/av3J*^ mice were killed at P3 by decapitation and fixed using 4% PFA for 2 h at room temperature.

#### Immunofluorescence

The fixed cochleae were washed in PB, dissected into cochlear spirals, permeabilized with 0.5% Triton X-100 in 0.01M phosphate-buffered saline (PBS) at pH 7.4 for 30 min at room temperature, immersed in 10% goat serum (GS) in PBS for 1 h to block non-specific labelling, and incubated for 48 h at 4°C in anti-LHFPL5 antibody diluted 1:100 in 1% GS-PBS. The long incubation time was based on preliminary experiments and used to ensure maximal labelling. After rinsing in 1% GS-PBS, they were incubated for 2 h at room temperature in a mixture of Alexa Fluor 568 goat anti-rabbit IgG (H+L) (diluted 1:50) and phalloidin-FITC (diluted 1:250–1:500) in 1% GS-PBS and washed in PBS. In experiments where we wished to quantify fluorescence, phalloidin was left out, to avoid red-green channel cross talk. The labelled cochlear segments were mounted in ProLong Diamond Antifade Mountant (P36965; Life Technologies, USA) or our own custom anti-fade solution (10 μM *p*-phenylenediamine in 30% polyvinyl alcohol and 30% glycerol) with coverslips and viewed under a 100 X oil-immersion objective using a BioRad MRC 1024 confocal laser scanning microscope.

#### Pre-embedding immunogold labelling

The fixed cochleae of CD/1 and *Lhfpl5*^*-/-*^ mice were washed in PBS, dissected into cochlear spirals, permeabilized with 0.5% Triton X-100 in PBS for 30 min at room temperature, immersed in 10% GS-PBS for 1 h at room temperature to block non-specific labelling and incubated for 48 h at 4°C in anti-LHFPL5 antibody diluted 1:100 in 1% GS-PBS. After rinsing in 1% GS-PBS, they were incubated in goat anti-rabbit IgG conjugated to 10 nm gold particles for 2h at room temperature and washed in PBS. They were then fixed in 2.5% GTA in 0.1M NaCac (pH 7.4) for 2h, washed in buffer, fixed in 1% osmium tetroxide/NaCac for 1h, dehydrated through a graded series of ethanol (70%, 80%, 90%, 100%, dry 100%) for 30 min in each concentration, infiltrated in increasing mixture of ethanol and Spurr resin, followed by pure Spurr resin which was polymerised at 60°C overnight. Semithin and ultrathin radial sections were cut from the apical and basal regions of the cochleae using a Leica Ultracut UCT ultramicrotome (Solms, Germany) and collected on 200-mesh thin-bar copper grids. Semithin sections were examined unstained whilst ultrathin sections were stained in 2% ethanolic uranyl acetate for 20 min and lead citrate for 5 min. The sections were then examined using a JEOL JEM 1230 transmission electron microscope operated at an accelerating voltage of 100 kV.

#### London Resin (LR)-White embedding and post-embedding immunogold labelling

For post-embedding immunogold labelling, samples were embedded in LR-White resin. Dissected apical halves of fixed cochleae from *Lhfpl*5^+/-^ and *Lhfpl*5^-/-^ at P5 were washed in 0.1 M PB and then embedded as segments. Whole fixed cochleae at P12 from *Lhfpl*5^+/-^ and *Lhfpl*5^-/-^ and P14 from CD/1 were washed in 0.1 M PB followed by distilled water, and required to be decalcified for embedding; this was done using 5.5% ethelenediaminetetraaceticacid (EDTA) containing 0.1% PFA for 3 days at 4°C. Both the P5 and the P12, P14 cochleae were then dehydrated in graded series of ethanol (70%, 80%, 90%, 100%, dry 100%) for 30 min in each concentration, infiltrated with LR-White resin (Agar Scientific), and polymerised in LR-White at 50°C overnight. Once embedded, the whole P12, P14 cochleae were microsliced in the midmodiolar plane.

Ultrathin sections were cut from the embedded samples using a Leica Ultracut UCT ultramicrotome and collected on glue pen-coated 200-mesh thin-bar nickel grids. The grids containing sections were washed in 0.05 M Tris-buffered saline (TBS), pH 7.4, non-specific labelling blocked with TBS containing 20% GS and 0.2% Tween 20 (GS-T20-TBS) for 30 min at room temperature, and incubated overnight at 4°C in anti-LHFPL5 antibody diluted 1:50 in TBS containing 1% bovine serum albumin and 0.2% Tween 20 (BSA-T20-TBS). The sections were then washed in BSA-T20-TBS (3 X 10 min), non-specific labelling blocked in GS-T20-TBS for 15 min, incubated for 2h at room temperature in goat anti-rabbit IgG conjugated to 10 nm gold particles diluted 1:20 in BSA-T20-TBS, washed in TBS followed by distilled water, stained in aqueous 2% uranyl acetate for 20 min and examined using a JEOL JEM 1230 transmission electron microscope operated at an accelerating voltage of 100 kV.

#### Quantification of immunolabelling

Immunoflurescence labelling was quantified by using a fixed area marquee tool in Adobe Photoshop ® of 10 X 10 pixels placed over individual OHC bundles. A total of 10 bundles were used; only those where the optical section plane lay immediately above the cuticular plate were evaluated.

Immunogold labelling was quantified on both pre- and post-embedding samples. To analyse the distribution of labelling in the bundle, about 50 images was assessed and gold particles were scored according to the position of the particle in bins of 0.1 μm width above the cuticular plate. Gold particles on the apical membrane were also counted. Where possible, the positions of the tips of ranked stereocilia were also scored, although for immature bundles, it was not possible to do this accurately.

For the developmental progression of expression, pre-embedding labelling was used from equivalent regions of the apex and base at different ages. Total particle number per image was determined, and the number of images depended on the orientation of the bundles, to ensure that as much of the labelling as possible was captured. Approximately 50 images were evaluated for one cochlea from each of three animals per time point. Sections were taken at a position of 180^0^ from the apex (approximately 80% of the full length of the organ of Corti from the basal end). We also evaluated the number of tips labelled at different ages, and the number of particles per labelled tip. For apical/basal comparisons, sections were cut and analysed from the basal turn as far along the cochlea as possible. To assess kinocilium labelling, because the profiles were varied, we determined gold particle number per area of the profile in all images where the kinocilium was present. The homozygote and heterozygote protocadherin 15 deficient mice (n = 3 for each) were compared by (i) evaluating the particle number as a function of height above the cuticular plate, and (ii) counting the proportion of particles over unranked stereocilia as a function of total labelling of the bundle in 20 OHC images per sample after pre-embedding labelling. Data were compared statistically using the Wilcoxon (Mann-Whitney) test, *p* < 0.05 taken as significantly different.

## Results

### Maturation of the hair bundles

A detailed account of hair bundle morphogenesis is not intended here, but in order to understand the structural context of the labelling patterns, we provide SEM images of different ages. The hair bundles in the apical turn of the cochlea are quite immature at P0 and gradually develop to become mature by P12. This picture is made more complex by the fact that maturation is also more advanced in hair bundles in the basal turn of the cochlea at each developmental age. In the following description therefore, we have concentrated on a location at approximately 80% of the length from the basal end of the organ of Corti, from where the majority of the data reported in this study have been obtained.

**Fig 1:**
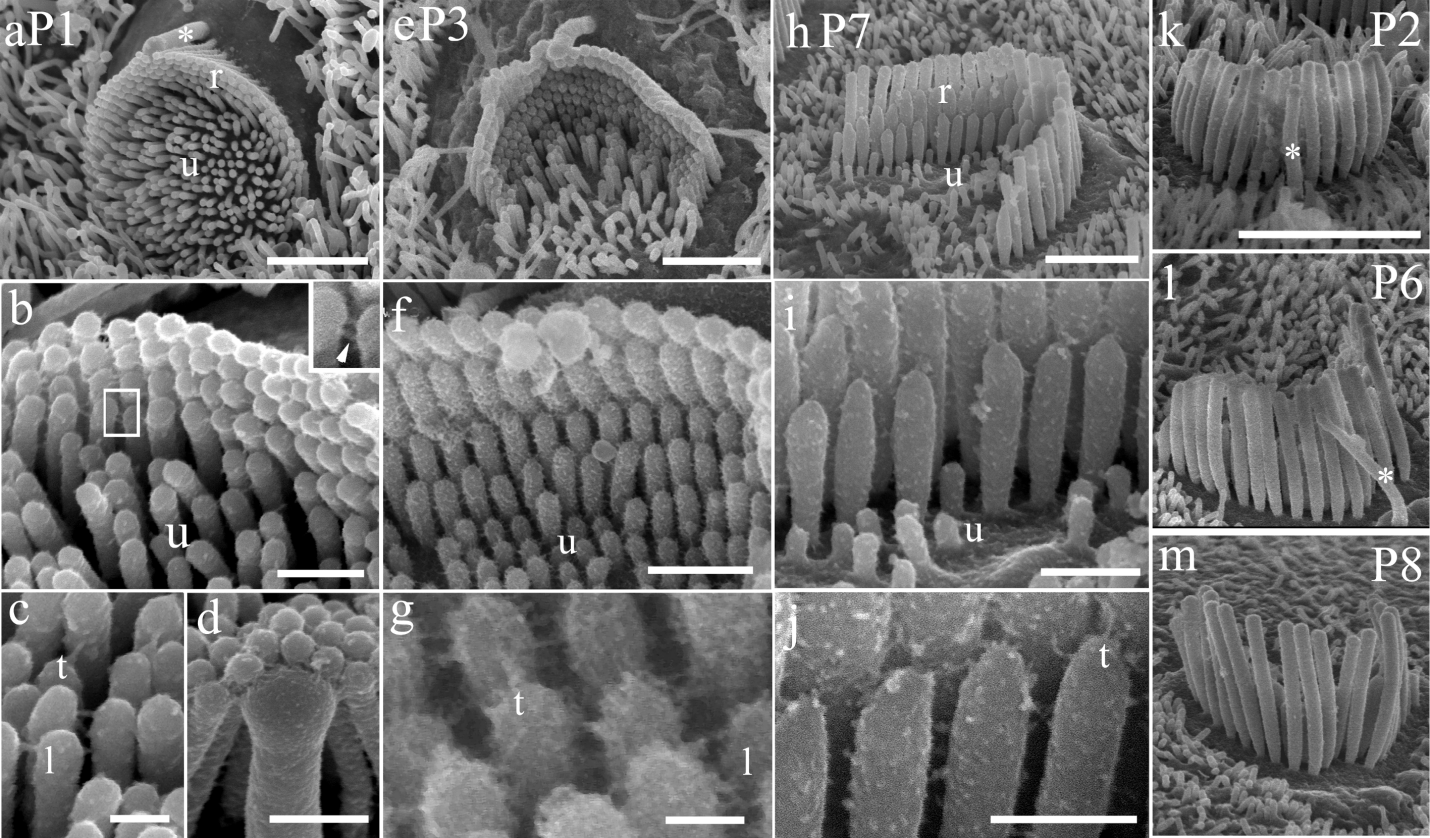
SEM of representative stages of hair bundle development in CD/1 mouse OHCs at approximately 80% of the distance from the cochlear base. P1 (a–d), P3 (e–g) and P7 (h–j) are illustrated. (a) At P1 bundles initially consist of large numbers of thin unranked stereocilia (u), similar in width to adjacent supporting cell microvilli, and the beginning of ranked stereocilia (r). (b) There are four to five ranked rows, with lateral links between the ranked stereocilia (inset, arrowhead). c) At higher magnification, tip links can also be seen in the ranked rows (t) and lateral links between unranked stereocilia (l). (d) The kinocilium is also linked to the tall stereocilia, most clearly at P1. (e) By P3 both ranked and unranked stereocilia become thicker with short unranked stereocilia gradually becoming reduced in number. (f) Several ranked rows (~6) become apparent behind unranked (u) stereocilia. (g) The unranked stereocilia have lateral links (l), and numerous tip links (t) are seen, sometimes more than one per tip, on the ranked rows. (h) By P7, most of the unranked (u) stereocilia and some of the short, ranked rows have gone, leaving the almost adult pattern of three ranked rows (r). (i) The lateral links have become reduced. (j) Multiple links are still seen around the tips. (k–m) The kinocilium (*) shows gradual thinning between P2 and P6, and is mostly absent by P8 at this location. Scale bars: a, e, h = 5 μm; b, f, i = 1 μm; c, d, j = 500 nm; g = 100 nm; k–m = 10 μm.

By SEM, outer hair cells (OHCs) at P0—P1 (Fig 1A–1C) can be seen to possess many short microvilli-like stereocilia of approximately equal height (hereinafter referred to as ‘unranked’), similar in width to the adjacent supporting cell microvilli, and a few taller stereocilia, of a similar thickness, beginning to form the ranked rows characteristic of the adults (hereinafter referred to as ‘ranked’). At P3 the ranked stereocilia become more prominent, but there are still short unranked stereocilia in the center of the bundle (Fig 1E–1G). These are now distinct from microvilli as they are thicker, shorter and less bent over than the latter, as displayed on adjacent supporting cells. The ranked region consists of rows of stereocilia lined up in the hexagonal arrangement typical of adult hair cells, increasing in height across the bundle towards the tallest row. However, there are extra rows of shorter stereocilia amongst the ranked group at early stages, when compared to the adult pattern of three ranked rows of short, intermediate and tall stereocilia that are evident by P7 (Fig 1H–1J). By P7, the OHC have a small number of unranked stereocilia in the center of the apex but these are absent in adults (see [16]).

The extracellular links connecting stereocilia also show changes as the bundles mature (see also [19]). Multiple links at the tips (hereinafter referred to as ‘tip link-like links’) and lateral links are found amongst the ranked rows at P0—P1 (Fig 1C), but there are often extra links at the tips at the early stages up to at least P7 in the cochlear apex (Fig 1G and 1J). Unranked stereocilia are also interconnected by lateral links, but a specific tip link cannot be defined because of the lack of ranking. These links become gradually diminished and are no longer detectable at P7 in the apical location (Fig 1I) and the unranked stereocilia themselves are diminished in size until they are completely lost. Lateral links are gradually reduced in quantity until in the adult they consist of top connectors near the tips of the shorter stereocilia and lateral links along the shafts but no links in the ankle region ([19] and see review [20]).

The kinocilium, which in immature cells is located behind the tallest row of stereocilia, approaches the tall stereocilia at an opposing angle to that of the ranking (i.e. in the negative direction); typically, only the topmost portion of the kinocilium is in contact where it forms links to the tallest row of stereocilia (Fig 1D). The kinocilium is absent or reduced to a tiny stump in the bundles by P8 at this location (Fig 1K–1M).

### Immunofluorescence evaluation of LHFPL5 distributions in wild-type and *Lhfpl5*^-/-^ mice

**Fig 2:**
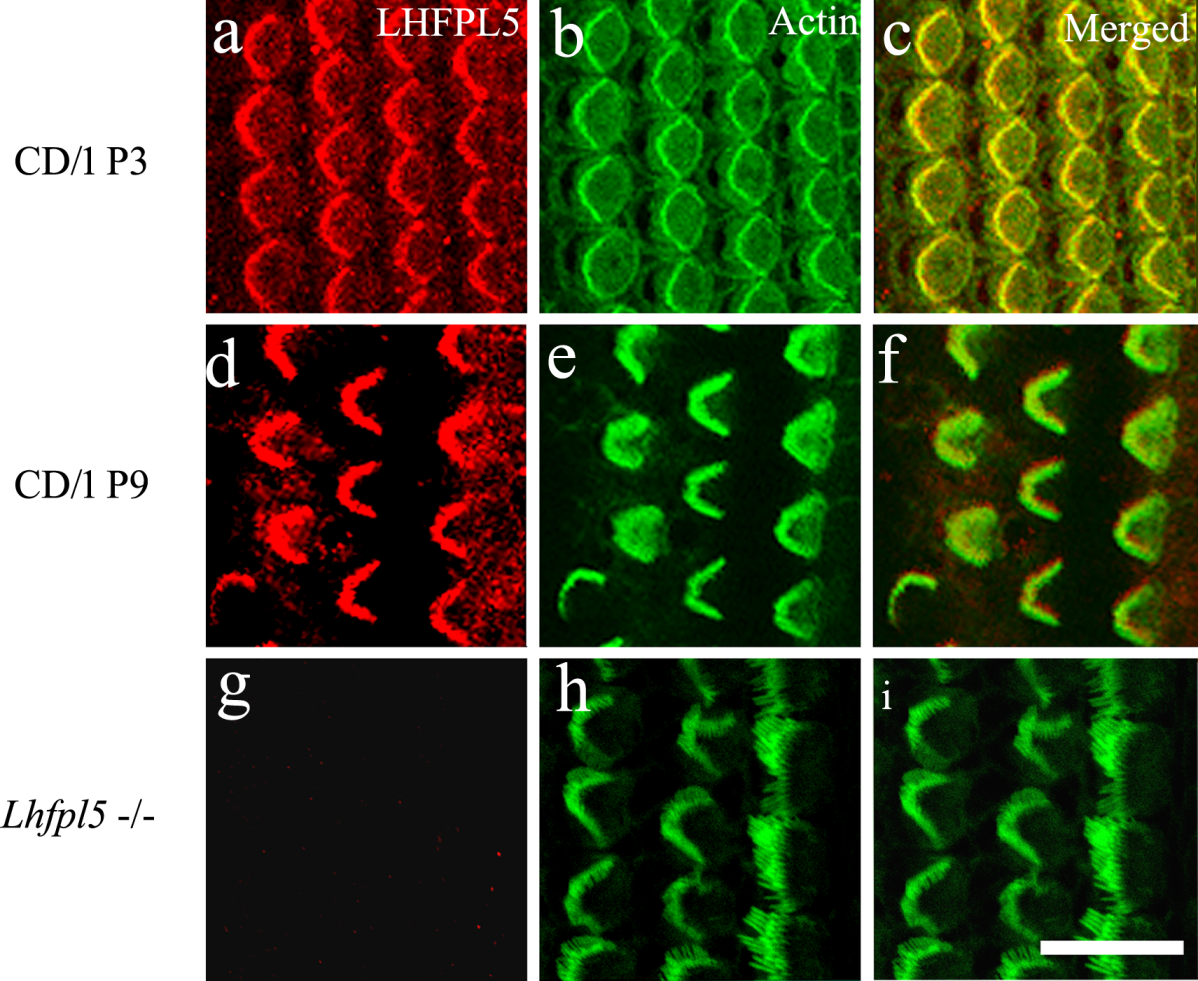
Confocal images of immunofluorescence labelling for LHFPL5 (red) and phalloidin (green) in CD/1 mice at approximately 80% of the distance from the cochlear base. Upper row, P3; second row P9, and bottom row *Lhfpl5*^*-/-*^, P5. Both IHC and OHC hair bundles are positive for LHFPL5 at both ages. Some labelling is also detected on the apical surface, especially in CD/1 P3. The knockout shows an absence of bundle labelling. Scale bar = 20 μm.

Immunofluorescence and confocal microscopy were used to establish positive labelling patterns in wild type CD/1 (P0 –P21) and C3HeB/FeJ (P9)) mice. Similar data were obtained from both. Labelling confirmed robust expression of LHFPL5 in the hair bundle at least to P9 (Fig 2A–2F). At P3, LHFPL5 appeared to be expressed over much of the apical region, including the v-shaped hair bundle and consistent with labelling over the unranked stereocilia although they could not be resolved. Labelling in the kinocilium was not detected by immunofluorescence. At P9, LHFPL5 expression was largely restricted to the V-shaped hair bundle. The labelling appeared to be specific as it was absent in *Lhfpl*5^-/-^ mice (Fig 2G–2I).

**Fig 3:**
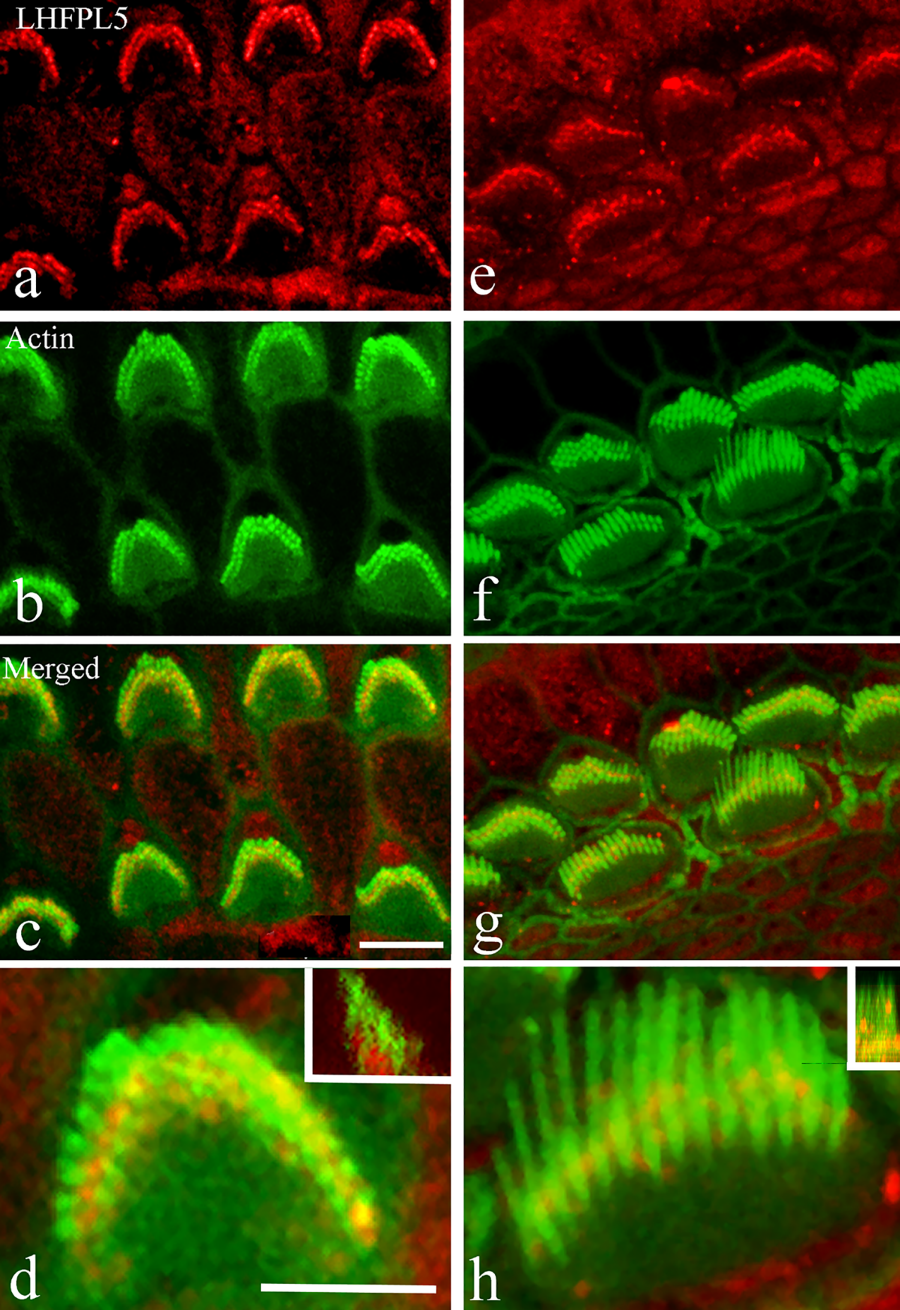
Confocal images of immunofluorescence labelling for LHFPL5 (red) and phalloidin (green) at P9 in C3HeB/FeJ mice at approximately 80% of the distance from the cochlear base. (a–d) distinct red fluorescence is associated with the OHC stereocilia and (d) reveals that a significant proportion of the signal lies between the stereocilia. The inset in (d) illustrates an orthogonal cut plane, indicating that most of the LHFPL5 fluorescence is in the lower part of the bundle. (e–h) Equivalent images of the IHCs show similar distribution patterns. Most of the labelling is between or on the shorter ranks and not the tallest row of stereocilia. The inset in h shows an orthogonal view similarly indicating most of the colocalised (orange) signal is in the lower part of the bundle. Scale bars: a–c, e–g = 10 μm; d, h = 5 μm.

When examined in more detail at P9, LHFPL5 was localised to the tip regions of stereocilia, as indicated by a yellow colocalisation signature, but also to a degree distinct from the stereocilia lower down the bundle in both OHCs (Fig 3A–3D) and IHCs (Fig 3E–3H). Using the cut plane or orthogonal view function of the confocal microscope software revealed that the majority of labelling was near the base of the bundle (Fig 3D and 3H).

### Immunogold TEM evaluation of changes in LHFPL5 distribution during maturation

Immunogold labelling followed by TEM was used to localise more precisely LHFPL5 in the OHC bundle from P0-P21. Pre-embedding immunogold labelling (where tissue is labelled prior to resin embedding) is more sensitive than the alternative technique (post-embedding immunogold labelling) but can suffer from lack of penetration of gold particles into protein-rich areas (see discussion in Hackney and Furness[20]). We therefore used both techniques to provide a fuller description of the distribution of LHFPL5 as the hair bundles mature.

The distribution of labelling was evaluated during bundle development from P0 to P21 in CD/1 mice using semi-thin sections (250 nm) and ultrathin sections (80–100 nm) of pre-embedding labelled cochlea. We confirmed salient points of these distributions in *Lhfpl5*^*+/-*^ at P5 and P12, and CD/1 at P14, using ultrathin sections and post-embedding labelling, which also provided additional data.

**Fig 4:**
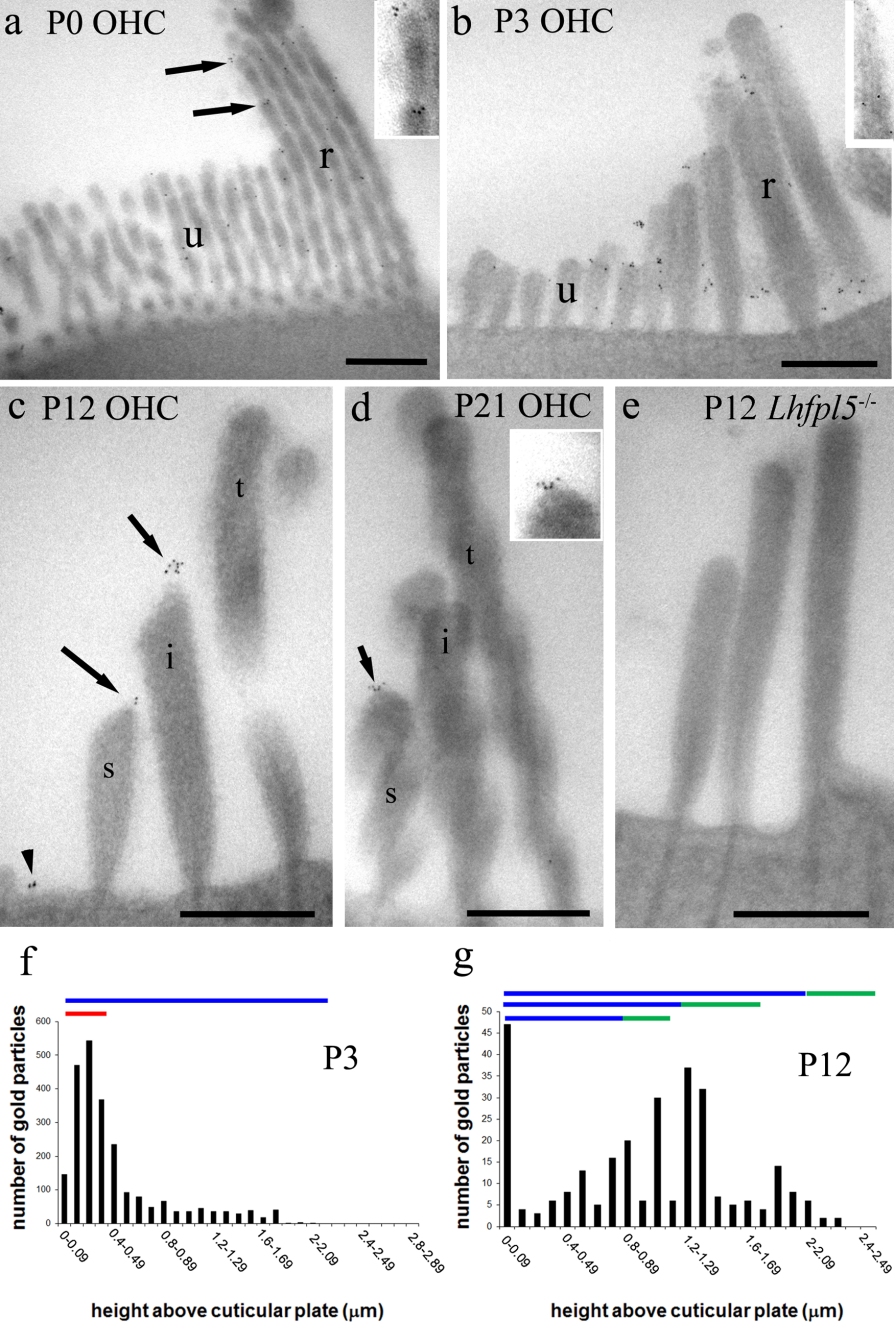
Semi-thin sections of pre-embedding immunogold labelling of OHC bundles from P0 to P21 at approximately 80% of the distance from the cochlear base. (a) At P0, labelling is distributed throughout the unranked (u) and developing ranks (r) of stereocilia, and at the tips of ranked stereocilia (arrows, region enlarged 2X in inset). (b) At P3, labelling is associated with lower shaft ankle/lateral links of the stereocilia and the tips of mainly shorter and intermediate ranked stereocilia, and along the kinocilium. The kinocilium is also labelled (inset, same magnification). (c) At P12 there are three ranked rows (s = short, i = intermediate and t = tall) and very few unranked stereocilia. Labelling is confined mostly to the tips of short and intermediate ranked stereocilia. Note that some tip labelling appears displaced (see Discussion for a consideration of this effect). Apical membrane labelling is also visible (arrowhead). (d) At P21 there are still particles labelling on some tips (arrow and inset, X1.5 enlargement). (e) No labelling is detectable in the *Lhfpl5*^*-/-*^ at P12. (f) Plot of the distribution of particles according to height from the apical membrane/cuticular plate in P3 hair bundles. The height of ranked (blue) and unranked (red) stereocilia is indicated by the horizontal lines. (At this stage, determining which of the ranked rows a stereocilium belongs to is difficult because of the minimal differences in height and the extra rows). The counts show gold particles towards the ankle/lower shafts and labelling of the apical membrane. (g) By P12, the distribution of particles is much more towards the tips (indicated by green horizontal bars) of the short and intermediate ranked rows (blue horizontal bars). Scale bars: 500 nm.

At P0 gold particles were localised to the many thin unranked stereocilia and to those beginning to form ranks in OHCs (Fig 4A). Labelling at this time point could also be found at the tips. By P3, when defined ranks of stereocilia are more evident, labelling occurred at the tips of ranked stereocilia of OHCs, including the tips of tallest row. There was also labelling along the lower shafts of the stereocilia, in association with lateral links, occasionally on the shaft of the kinocilium, on either side, and also on the unranked stereocilia, consistent with the confocal data (Fig 4B). By P12, the labelling of the tips of the tallest stereocilia and shaft labelling was diminished concomitantly with a reduction in lateral links by this stage, but tip labelling of the short and intermediate rows remained (Fig 4C). This labelling tended to be localised where the lower part of the tip link is located, although some separation from the tips also sometimes occurred. The latter may be due to difficulties in defining precisely the tip in our unstained semithin sections, or the tips being present in adjacent section, or by displacement of particles during the lengthy procedures. The gold particles appear to trace tenting of the membrane on the shorter and intermediate stereocilia (Fig 4C). By P21, tip labelling in shorter rows could still be detected but fewer tips were labelled (Fig 4D) (see below). In *Lhfpl5-/-* animals, virtually no labelling was observed in the bundle at P12 (Fig 4E).

Quantification of the distributions was performed by counting gold particle location along the length of the stereocilia above the cuticular plate. This showed a shift in particle distribution from significant quantities over the lower shafts of the stereocilia at P3 (Fig 4F) to predominantly tip labelling at P12 (Fig 4G) and there was also some apical membrane labelling (Fig 4C) as noted in the lowest bin of the histograms (4f, g). We assessed whether there was any regional distribution of the apical membrane labelling by analysing the number of particles on the membrane in the kinociliary side vs the non-kinociliary side at P3. Counts from approximately 50 images from each of three samples revealed that at P3, the labelling was stronger on the membrane on the kinociliary side of the bundle (mean 13±2.4 particles per sample) than on the opposite side (6.7±2.3 particles per sample), thus greater by a factor of approximately 2:1.

**Fig 5:**
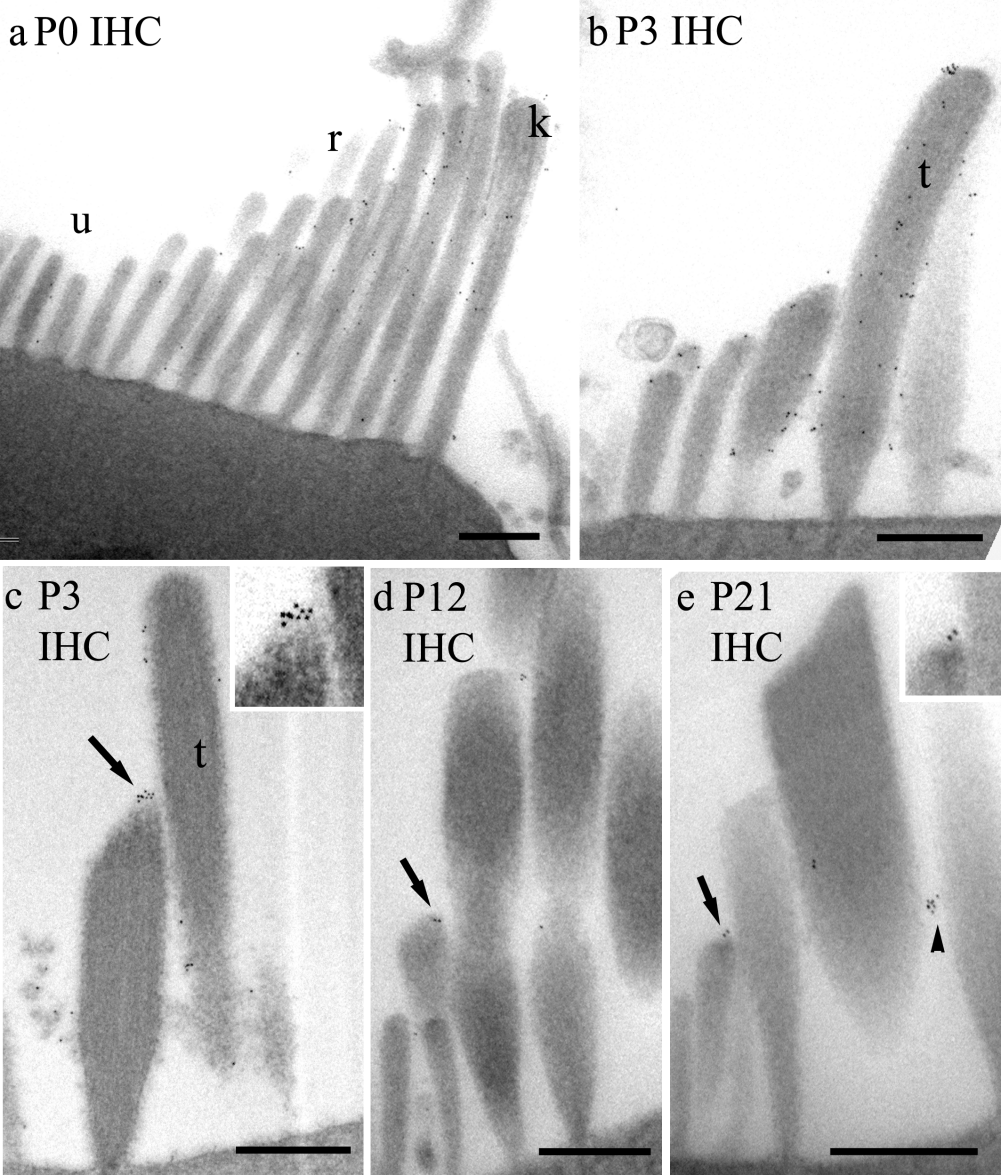
**(a-e) Pre-embedding immunogold labelling of P0 to P21 IHC bundles at approximately 80% of the distance from the cochlear base.** (a) At P0 some labelling is found on unranked stereocilia, but more is found over the developing ranked stereocilia and some can be seen on the kinocilium (k). (b) P3 semithin and (c) P3 ultrathin sections. Gold particles are seen along the shafts and tips at P3, including the tall stereocilia (t). The tip labelling is seen at 1.75X enlargement in the inset in c. (d) By P12 and (e) P21, only weak labelling is visible on the shafts of the stereocilia (arrowhead) but the tips of shorter rows of stereocilia are still labelled (arrows; inset X2). Scale bars = 500 nm.

In IHCs, the bundle development and the associated labelling pattern was mostly similar to the OHCs, with a minor difference: in sections of the P0 IHC, the proportion of gold particles was greater over the developing ranked than the unranked stereocilia. The kinocilium was also clearly labelled, as in OHCs, with particles located along the shaft and not confined to the base or to one side (Fig 5A). By P3, it was distributed mostly on the lower shafts and the tips of the ranked stereocilia (Fig 5B and 5C). By P12 it had been refined primarily to the tips (Fig 5D) but with some on the shafts, which was similar at P21 (Fig 5E).

**Fig 6:**
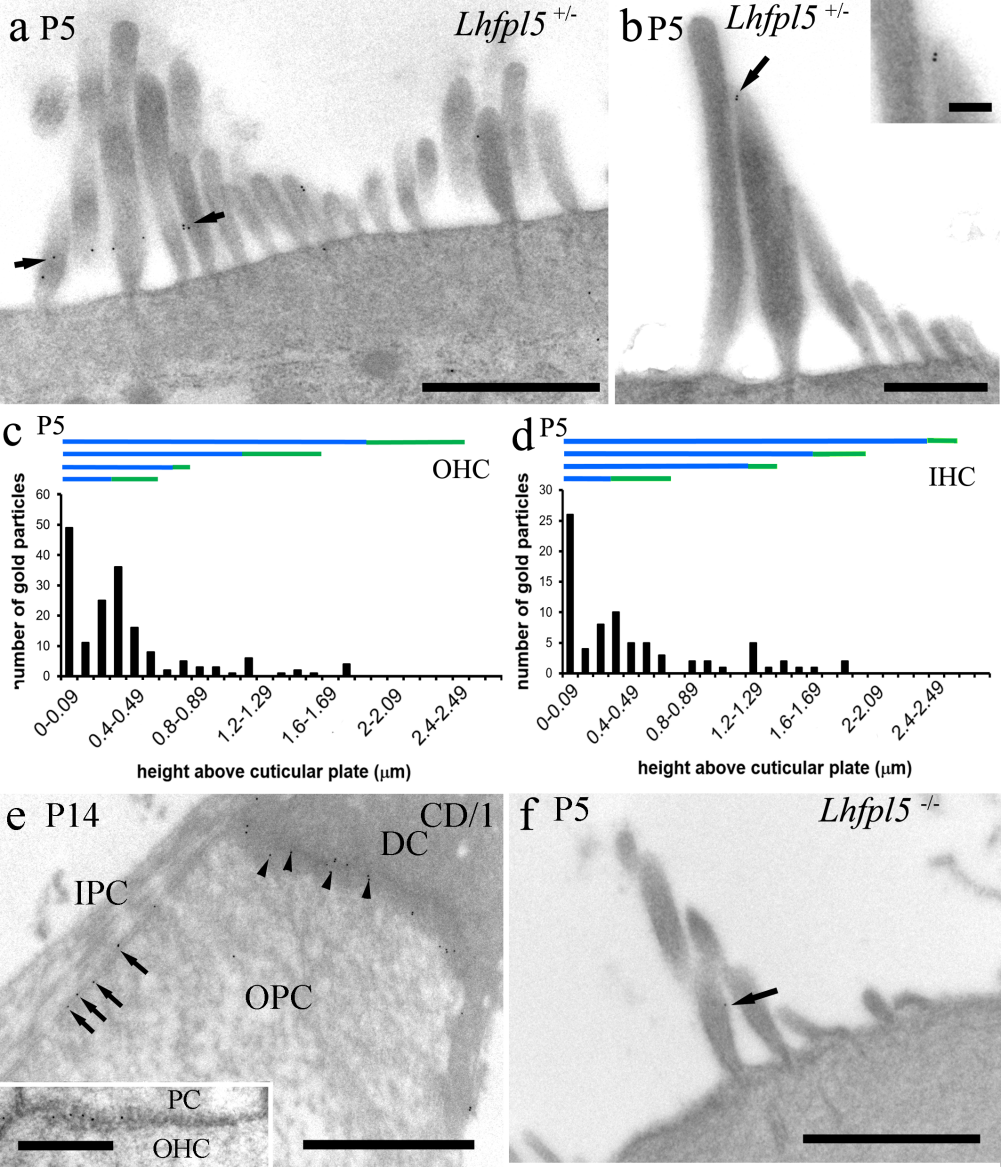
Postembedding immunogold labelling at approximately 80% of the distance from the cochlear base. (a-d). Post-embedding immunogold labelling of *Lhfpl5*^*+/-*^ at P5 confirms distributions seen between P0 and P9 in pre-embedding labelling of wild-type mice. (a) Lower shaft labelling (between arrows) and (b) tip labelling (arrow and inset) were both detected. (c, d) Counts from OHCs and IHCs revealed a tendency to label the lower parts of the bundle, but there was detectable enrichments associated with the tips (horizontal green bars) of all except the tallest stereocilia. (e) At P14 in CD/1 mice, labelling was also detected in the junctions between pillar cells (e.g. arrows) and also between pillar cells, Deiters’ cells (e.g. arrowheads) and hair cells (inset), which was not detected in pre-embedding, due probably to steric hindrance. IPC = inner pillar cell, OPC = outer pillar cell, PC = pillar cell, DC = Deiters’ cell, OHC = outer hair cell. (f) Minimal labelling was detected in *Lhfpl5*^*-/-*^ mice–the only particle observed in 50+ sections is shown (arrow). Scale bars: a, e, e inset, f = 1 μm, b = 500 nm, b inset = 200 nm.

With post-embedding labelling of *Lhfpl5*^*+/-*^ (heterozygote) mice at P5, the pattern was very similar to P3 pre-embedding wild-type labelling with lower shaft and tip labelling evident (Fig 6A and 6B). The advantage of the post-embedding method is it allows better antibody penetration into regions of denser material, such as protein rich areas, than pre-embedding. However, it was harder to see the tip labelling presumably because of the reduced number of antigenic sites available in thin section instead of those in the full stereocilium tip, and it has lower sensitivity. Nevertheless, gold particle counts made against height above the cuticular plate revealed similar patterns as the pre-embedding. Labelling was present in the lower parts of the bundle, but also some tip enrichment was observed, excluding the tallest row (Fig 6C and 6D). Labelling was not detected on the kinocilium, but this may reflect the fact that few profiles of this structure are seen in ultrathin sections. At P12 or P14 very little labelling was detected by post-embedding in the hair bundle. Similar patterns were also observed in IHCs, comparing P5 and P12 (*Lhfpl5*^*+/-*^) or P14 (CD/1).

In addition, however, post-embedding revealed evidence of labelling in the junctions between hair cells and supporting cells, between pillar cells and Deiters’ cells, and between adjacent pillar cells (Fig 6E). In post-embedding labelling of the *Lhfpl*5^-/-^ mice at P5, there was much reduced labelling in all the regions noted above, i.e. the stereocilia (Fig 6F) and junctions indicating that the labelling observed in the wild type was specific.

### Quantitative changes in distribution of LHFPL5 in CD/1 mice during development

**Fig 7:**
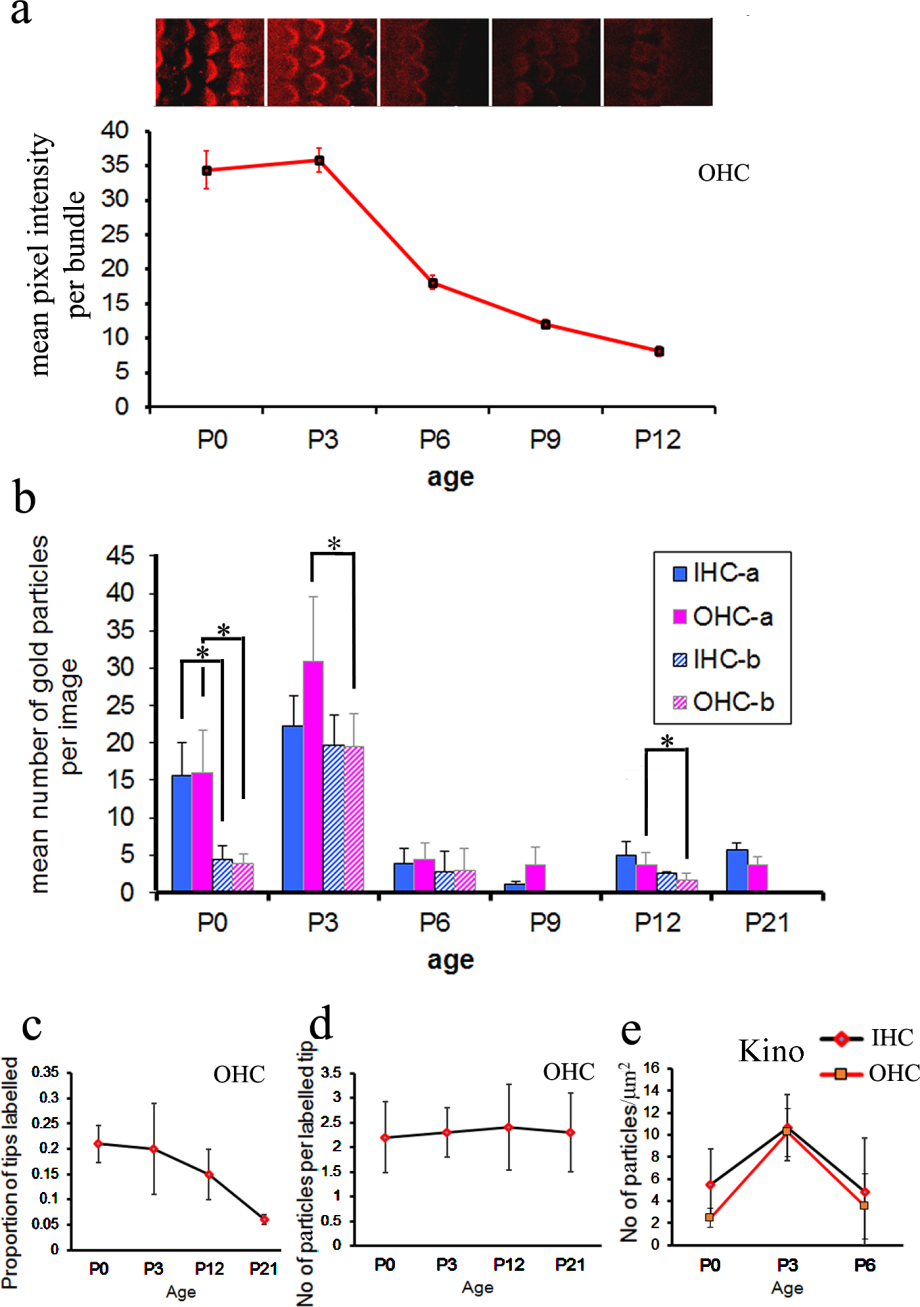
Quantification of immunofluorescence and immunogold labelling from P0 to P21. a. Panel of confocal images of OHCs from the CD/1 mouse cochlea at different ages, acquired under identical conditions in the same experiment at approximately 80% of the distance from the cochlear base. Labelling was performed without phalloidin in order to eliminate the possibility of cross-talk between red and green channels in the confocal. Fluorescence intensity is high at P0 and P3 and drops after P3. No labelling was detected after P12. A graphical representation of the changes is shown below, derived from fluorescent intensity measurements of hair bundles from each of the above stages. Error bars represent the S.E.M of mean intensity measured in 10 bundles from two images. (b) Quantitative analysis of immunogold labelling in apical (at approximately 80% of the distance from the cochlear base) and basal (approximately 20–30% from the cochlear base, where possible), representing the mean of 3 different animals at each age. Counts reveal a similar high level of expression in P0 to P3 in both IHCs and OHCs, dropping substantially to a similar level over P6 –P21. Throughout development, apical labelling is higher than basal labelling, but not always significantly. Significant differences at *p* < 0.05, Wilcoxon (Mann-Whitney) test, are indicated by asterisks. No data could be obtained for P9 or P21 basal rgions. (c) Developmental changes in the proportion of tips that are labelled. The proportion is highest at P0 and P3 and then decreases with maturation. (d) Developmental changes in the number of particles per labelled tip. The number on each labelled tip does not change from P0 to P21. (e) Analysis of kinocilium labelling (particles per μm^2^) on both IHCs and OHCs reveals a peak at P3 compared with P0 and P6.

The fluorescence and gold particle labelling also allows us to quantify the spatiotemporal pattern of LHFPL5 expression in the bundle. To quantify the expression pattern we collected immunofluorescence images of OHCs at P0, P3, P6, P9, P12 and P20. The imaging conditions were maintained constant and two images recorded from the apical region in each sample for quantification. Five complete bundles were then assessed for image intensity in each image and this was then plotted against age (Fig 7A). This showed about the same levels of expression at P0 and P3 and then a gradual decrease to P12 after which no expression could be detected.

The pre-embedding immunogold labelling used for qualitative evaluation was also assessed quantitatively. Three cochleae from different mice at each age were used in which total labelling of the bundle was assessed by counting gold particles in approximately 50 images.

The gold particle analysis of apical hair bundle labelling was mostly consistent with the results from the immunofluorescence at the same location, with IHCs and OHCs showing a peak in labelling at P3 reducing to relatively low levels by P6. A degree of labelling was retained even up to P21 in apical bundles (Fig 7B).

We also evaluated how many tips of ranked stereocilia were labelled at each age in OHCs, where the ranks are more easily defined than in IHCs, and how many gold particles were present per labelled tip (Fig 7C and 7D). The number of labelled tips at P0 and P3 was ~20% and whilst it was not possible to assign ranked stereocilia to specific rows at P0, at P3 more than half (51%) of labelled tips were the tallest stereocilia tips. By P12, the number of labelled tips dropped to 15% but now nearly all of the labelled tips (~90%) were in the shorter rows of stereocilia. At P21 the proportion of tips labelled dropped to 6%, 73% of which were shorter stereocilia tips. The number of particles per labelled tip was relatively constant from P0 to P21 (Fig 7D). Labelling on the kinocilium, which could not be unambiguously detected using confocal microscopy, was also assessed in IHCs and OHCs and showed a parallel change in labelling to the bundles (Fig 7E), but was virtually absent after P6 and the kinocilium itself was mostly absent by P8, as noted above.

### Apical-basal differences in LHFPL5 expression

As well as differences in temporal expression we examined spatial differences by comparing apical and basal hair cells at P0 to P12. The analysis of gold particle numbers per image revealed that the basal hair cells were always less heavily labelled than apical ones except in one mouse at P6 and only weak labelling was detected at P6 in the base. The apical-basal differences were significant for OHCs at P0,P3 and P12, and for IHCs at P0. No basal hair cells were evaluated at P9 and P21 due to dissection difficulties. Although basal hair cells had less expression of LHFPL5 than the same aged apical ones, the number of particles per labelled tip did not show a substantial difference either at P3 (2.27±0.47 apical vs 1.95±0.4 basal) or P12 (2.42±0.87 vs 2.57±0.46, respectively).

### Distribution of LHFPL5 in Pcdh15-deficient mice

The consistent patterns of labelling in pre-and post-embedding immunoelectron microscopy of the wild type suggest that, as expected from previous studies, tip enrichment of LHFPL5 is present, as predicted from its association with PCDH15 in the tip links. Thus, we decided to look at the distribution of labelling in *Pcdh15* ^*av3J/av3J*^ mice where tip link-like links are largely absent [8] and in the *Pcdh15* ^+*/av3J*^ heterozygote control.

**Fig 8:**
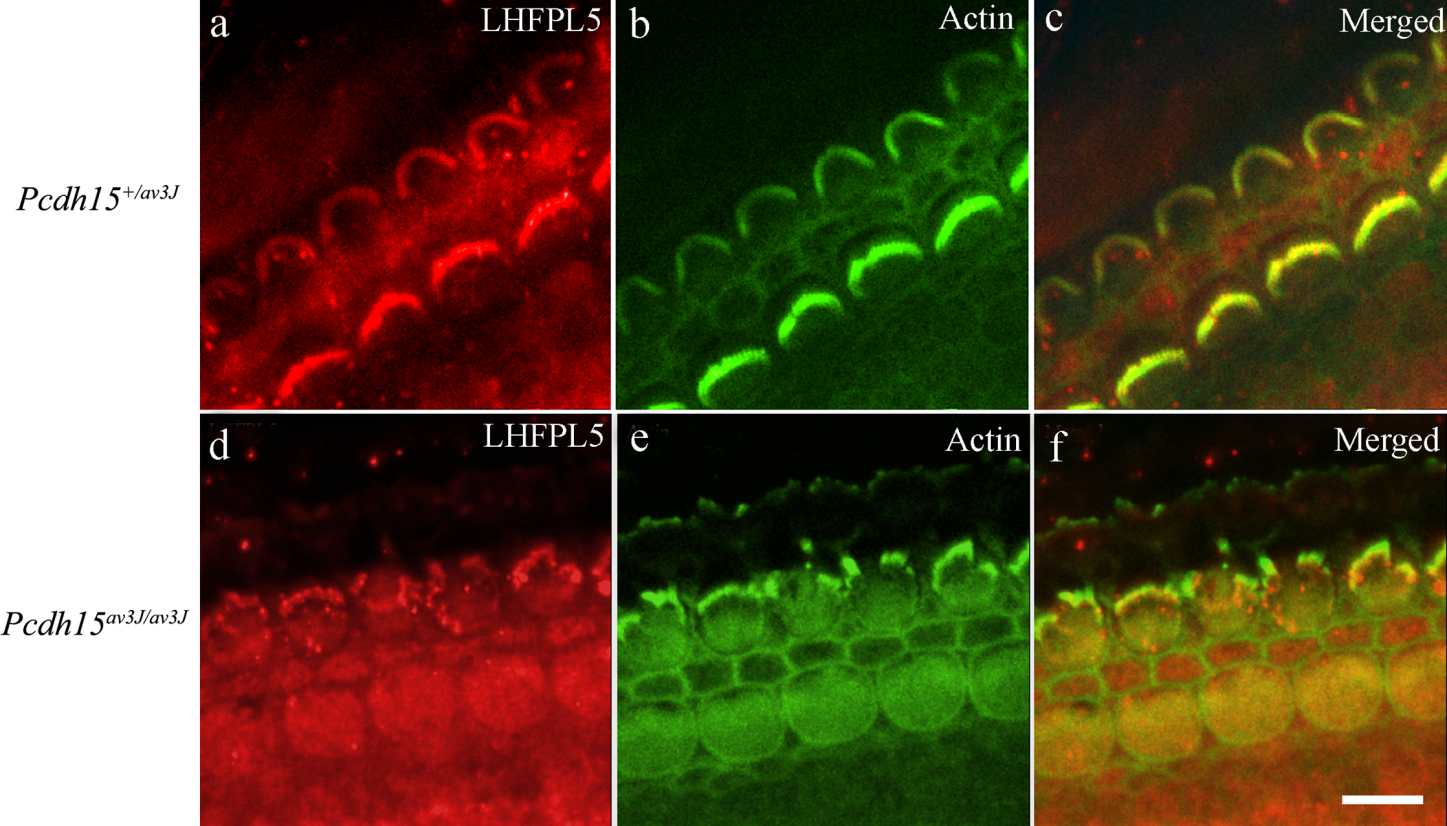
Confocal images of LHFPL5 (red) and actin/phalloidin (green) labelling in *Pcdh15*^*+/av3J*^ and *Pcdh15*^*av3J/av3J*^ mice at approximately 80% of the distance from the cochlear base. (a—c) In the heterozygote, labelling appeared similar to wild type. (d—f) In the homozygote, the phalloidin revealed that bundles were distorted, and the LHFPL5 could be found over the bundle and the cuticular plate region. Scale bars = 20 μm.

Using immunofluorescence at P3 the pattern of labelling of the heterozygote was similar to that of the wild type (Fig 8A–8C). In the *Pcdh15* ^*av3J/av3J*^ it was evident that there was still labelling for LHFPL5 in the hair bundles (Fig 8D–8F). However, no definitive tip labelling was observed and some of the fluorescence was located over the cuticular plate as well as on the lower portions of ranked stereocilia. We therefore performed pre-embedding immunogold labelling for more precise localisation.

**Fig 9:**
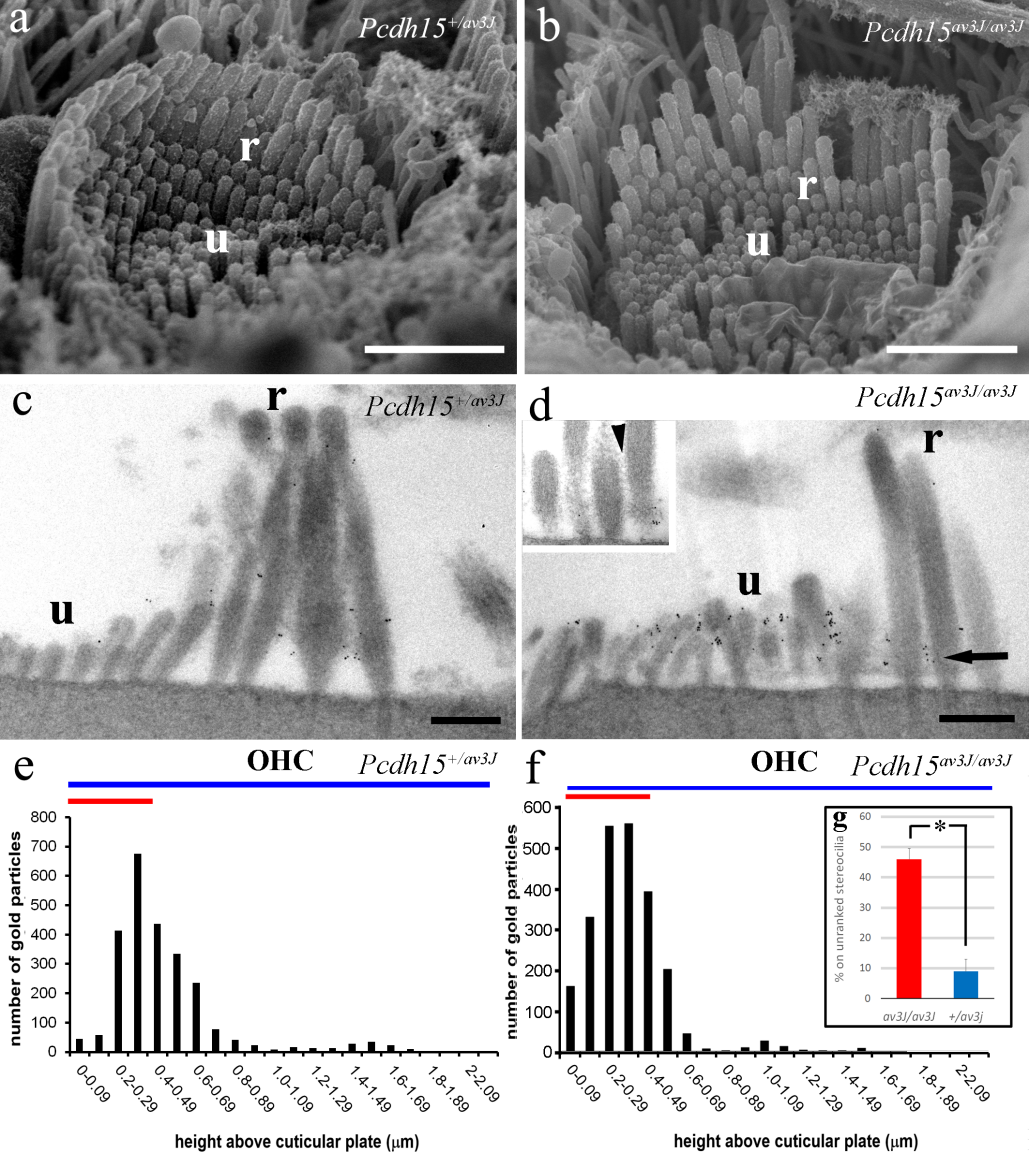
SEM and TEM of labelling in *Pcdh15*^*+/av3J*^ and *Pcdh15*^*av3J/av3J*^ mice. (a, b). SEM comparison of OHC hair bundles. The heterozygote is similar to wild type but the homozygote bundle is dysmorphic. Nevertheless the latter still has regions of ranked (r), as well as unranked (u) stereocilia. (c–d). Pre-embedding immunogold labelling using TEM in P3 heterozygote and homozygote hair cells at approximately 80% of the distance from the cochlear base. The heterozygote labelling resembles the wild type, but the homozygote shows predominant labelling on the unranked stereocilia. Note that lateral links occur between the unranked stereocilia, as well as the ranked stereocilia, and are still present in the homozygote (d, inset arrowhead, ultrathin section) where they are also labelled (arrow, main image). (e, f) Quantitative analysis of labelling on the heterozygote and the homozygote. Labelling is lower down the bundle in the homozygote compared with the heterozygote, and is more concentrated in the unranked stereocilia. The blue bars show the heights of the tall stereocilia, the red bar the heights of the ranked and unranked short stereocilia. (g). Relative proportion of total labelling over the unranked stereocilia in *Pcdh15*^*+/av3J*^ and *Pcdh15*^*av3J/av3J*^ mice OHCs. There is a significantly higher proportion on the unranked stereocilia in the homozygote, *p* < 0.05, Wilcoxon (Mann-Whitney) test. Scale bars: a, b = 1.5 μm; c, d = 500 nm.

By SEM, *Pcdh15* ^*av3J/av3J*^ P3 mice have hair bundles that are dysmorphic, being somewhat disorganised compared with the heterozygote (Fig 9A and 9B) and wild type controls (Fig 1E), but still possessing both categories of stereocilia: those that are ranked and those that are unranked. In addition, the kinocilium is located in an equivalent position to that in the heterozygote.

In the pre-embedding samples, labelling in the heterozygote was very similar to wild type P3, but in the homozygote, labelling was very much reduced on the tips of the ranked stereocilia, as might be predicted from the absence of tip link-like links. However, it was still present on the shafts of ranked stereocilia, in association with lateral links, and on the kinocilium. Furthermore, much of the labelling was now associated with the unranked stereocilia tips and sides (Fig 9D). To confirm this we, measured, as before, the distribution of labelling with height above the cuticular plate (Fig 9E and 9F). Compared with the heterozygote, labelling was distributed more towards the base of the bundle, confirming a higher level of labelling over the short, unranked stereocilia. This was not due to the absence of lateral links on the shafts, as they could clearly be seen in the images (Fig 9D, inset). There was also a small amount of apical membrane labelling in both, but this was not as pronounced as in wild type.

We analysed the proportion of labelling over ranked and unranked stereocilia in 20 images from three P3 *Pcdh15* ^*av3J/av3J*^ and *Pcdh15* ^+*/av3J*^ pre-embedding labelled mice cochleae each. This showed a substantial increase in proportion of gold over the unranked stereocilia in the *Pcdh15*-deficient mice from about 10% in the heterozygote to over 40% in the homozygote (Fig 9G).

## Discussion

Our main findings are: (i) LHFPL5 protein expression is present in hair bundles at P0, peaks about P3, then declines; (ii) LHFPL5 is initially localised throughout the hair bundle but becomes refined to the tips of predominantly shorter stereocilia by P12 (the onset of hearing), and is maintained in a small proportion of tips until at least P21; (iii) there is transient labelling associated with lower shaft/ankle links during development; (iv) there is a greater LHFPL5 expression apically than basally at each stage examined, but the amount of labelling at the tips in both locations is equal; (v) kinocilium labelling peaks at P3 then declines; (vi) in the absence of PCDH15, LHFPL5 is no longer localised strongly to the tips of stereocilia but is primarily found on unranked stereocilia and ankle links in P3 bundles; and (vii) there is expression in junctional complexes of the organ of Corti. These data appear to reflect true LHFPL5 distributions because they are absent in *Lhfpl5-/-* mice using all three labelling methods.

Our results add to the observations of Xiong et al. [13] who reported that labelling for LHFPL5 is associated with the tips of all rows of stereocilia, including the tallest row from P4-P7, with labelling substantially diminished after P7. We found labelling was still present by P9 using immunofluorescence, an observation that could be due to the longer antibody labelling times we used. We confirmed expression on the tallest stereocilia tips in early stages, becoming lost by P12. Xiong et al [13] also reported an absence of labelling from *av3J -/-* bundles whereas we observed it was present, although no longer at the ranked stereocilia tips. This difference may also represent longer antibody incubation times used. The detection of junctional expression in post-embedding labelling and not pre-embedding or immunofluorescence reflects the fact that in the former, antigenic sites are revealed after sectioning which occurs prior to labelling but potentially obscured in the latter two, where labelling is done first and hence could be limited by less effective antibody penetration.

### Refinement of LHFPL5 localisation coincides with maturation of the hair bundle and MET response

We here have examined the labelling in the context of bundle development. Initially short unranked stereocilia form the bundle [16] which then develops to its adult pattern by growth of several ranked rows along the kinociliary edge by P3, followed by reduction in the shortest of the ranked rows and loss of the unranked stereocilia on the non-kinociliary side especially between P3 and P7 at the apex; the base matures 2–3 days earlier. This is accompanied by refinement of tip link-like links to a single tip link by P12 and gradual reduction in other links. Loss of the ankle links during this period has also been reported [19]. The normal polarised transducer current activated by deflection of the hair bundle appears around P0 in the apical region and is half maximal by 2.5 d in apical OHCs and 2.0 d in IHCs. Prior to this, anomalous transducer currents occur but diminish by P2 [10].

In this context, LHFPL5 is present throughout the bundle, including at some of the tips of the stereocilia, at the onset of transduction (P0) and becomes refined to its adult location by P12, the onset of hearing, in two phases. In the first phase, P0 to P3, the more general distribution changes to primarily stereocilia tips and lower shaft/ankle links. Rising bundle labelling during this phase, coincides with increasing LHFPL5 mRNA expression [13]. In the second phase from P3 to P12 labelling is reduced and that of the tallest stereocilia tip is lost. The former is not consistent with the continued high mRNA expression to P25 [13]. Thus the mRNA expression data may not reflect strongly the actual level of protein present, a possibility noted in other studies [21,22]. Crucially, sustained tip labelling to P21 suggests LHFPL5 is a permanent component of the MET complex along with the tip links, despite the fact that not all tips are labelled (see below for discussion of this inconsistency). The tip labelling of shorter stereocilia confirms that LHFPL5 is located at the lower end of the tip link. Indeed, in some images, it appears to follow the tenting of the tip, consistent with the fact that the antigen is located in the C-terminus (cytoplasmic domain) of LHFLP5.

After P0, there is no diminution in the amount of labelling per *positive* tip amongst the shorter stereocilia, suggesting the full complement of molecules is established at least from birth onwards, although the number of tips labelled does decline, as will be discussed later. The association of LHFPL5 with PCDH15 has been suggested to mean that it is required for the proper targeting of the PCDH15 and TMC1 in the MET to the tips [13,14]. We suggest, however, that the association with LHFPL5 and these other proteins is interdependent, as although LHFPL5 is present in the bundle, there is loss of tip localisation of LHFPL5 concomitant with failure to form tip links in the protacadherin 15-deficient hair bundles [8].

However, it is puzzling that the overall proportion of detectable tips labelled is low, at P3 only 20%, and that this decreases subsequently; in comparison, the MET current is maximal in apical OHCs by P4 and does not decline at later ages. There may be several reasons for this. Firstly, since the antibodies recognise an intracellular part of the molecule, detergent treatment was used to permeabilize the membrane, which may be less effective at the stereocilia tips because of protein rich submembrane structures [20]. Secondly, when using immunogold, the particles may sterically inhibit penetration of the secondary antibodies. Thirdly, the weight of the particles may cause some loss or displacement during the elongated processing needed for TEM embedding. The latter could also explain why particles were not always directly on the tip of the stereocilia, although this displacement could also reflect the presence of the tip in a subsequent section. Fourthly, LHFPL5 may undergo some post-translational modification or protein binding that renders it less antigenic or accessible. These possibilities would reduce the efficacy of the labelling.

The association between different transducer complex proteins could explain why LHFPL5 molecules are present developmentally in locations other than the short stereocilia tips. PCDH15 is transiently present in the lateral links [6] and previous reports suggested LHFPL5 is absent from mouse hair bundles lacking PCDH15[13]. However, because we detected LHFPL5 in the latter, it must still be targeted somehow to stereocilia. Moreover, since the tall stereocilia tips also label at P3, this is difficult to explain by an interaction with PCDH15 or TMC1, because neither are present at the tips of the tallest stereocilia [4,6].

Our analysis suggests that in the P3 *Pcdh15* ^*av3J/av3J*^ bundles the LHFPL5 is on unranked stereocilia and on the shaft/ankle links. The situation in P3 *Pcdh15* ^*av3J/av3J*^ may be analogous to that of P0 wild-type hair bundles, perhaps before PCDH15 expression would normally occur, where unranked stereocilia show a substantial amount of labelling. This could indicate failure of the bundle to develop properly in the *Pcdh15* ^*av3J/av3J*^ mice. We also noted that lateral links were still present in these mice between both ranked and unranked stereocilia, although the PCDH15 is absent, indicating there are other components to these links that might interact with LHFPL5, for example VLGR1 [23].

We also found higher levels of overall bundle labelling in apical hair cells than basal hair cells. However, this difference does not apply to the stereocilia tips where the MET channel is located. There, the amount of LHFPL5 labelling per tip appears to be constant at every age and in both locations. This observations is consistent with there being the same number of MET channels (two) in both apical and basal OHCs [24].

### Is there a role for LHFPL5 in anomalous transduction currents?

In some circumstances, an anomalous mechano-sensitive current is activated by reverse deflection of the bundles, e.g. in the absence of tip links [8,9], or by sucking on the apical membrane[10]. Evidence now suggests that this stretch activated current is associated with PIEZO2 in early development and after tip-link loss [12]. It seems unlikely that LHFPL5 and TMC channels are associated with this conductance, as it is present in *Tmc1*^*-/-*^*;/Tmc2*^*-/-*^ mice. Nevertheless, apical membrane labelling for LHFPL5 was observed in our samples, and at P3 it was stronger behind the kinocilium with respect to the bundle, near the reported location for PIEZO2. No association between PIEZO2 and LHFPL5 is known, but if other MET components assembled with the LHFPL5 here, or in the unranked stereocilia where it also occurs, it is at least possible that MET channels could be activated in these locations.

The kinocilium was also labelled for LHFPL5. PCDH15 occurs in links between the kinocilium and the stereocilia in chicken utricular hair bundles (7), and TMC1 occurs in the kinocilium in mice (14). Hence the possibility remains that some aspects of the anomalous currents reflect altered distributions of these proteins during development and in the absence of tip links, but this is likely not the main explanation of these currents.

### Localisation to junctional complexes

The labelling we found associated with the junctional complexes suggests that LHFPL5 may associate with proteins there as well. This could reflect the fact that the junctions contain cadherins [25], so there is a possibility of a common binding site site that LHFPL5 attaches to on cadherins more generally.

## Conclusions

In conclusion, our data support a role for LHFPL5 in development of the transducer apparatus and show a gradual refinement in its localisation to the tips of the shorter stereocilia by the onset of hearing, consistent with down-regulation in places where it is not required. The presence of LHFPL5 at P21 is consistent with it being a component of the adult transducer apparatus. The data also suggest that in the absence of PCDH15, and therefore the tip links, mislocalization of components of the MET apparatus occurs.
